# YOLO-TARC: YOLOv10 with Token Attention and Residual Convolution for Small Void Detection in Root Canal X-Ray Images

**DOI:** 10.3390/s25103036

**Published:** 2025-05-12

**Authors:** Yin Pan, Zhenpeng Zhang, Xueyang Zhang, Zhi Zeng, Yibin Tian

**Affiliations:** 1 School of Computer and Information Science, Chongqing Normal University, Chongqing 401331, China; 2 College of Mechatronics and Control Engineering, Shenzhen University, Shenzhen 518060, China; 3 Department of Stomatology, The First People’s Hospital of Shunde, Southern Medical University, Foshan 528000, China

**Keywords:** root canal treatment, X-ray, void detection, deep neural network, YOLO, residual convolution, token attention, postoperative evaluation

## Abstract

The detection of small voids or defects in X-ray images of tooth root canals still faces challenges. To address the issue, this paper proposes an improved YOLOv10 that combines Token Attention with Residual Convolution (ResConv), termed YOLO-TARC. To overcome the limitations of existing deep learning models in effectively retaining key features of small objects and their insufficient focusing capabilities, we introduce three improvements. First, ResConv is designed to ensure the transmission of discriminative features of small objects during feature propagation, leveraging the ability of residual connections to transmit information from one layer to the next. Second, to tackle the issue of weak focusing capabilities on small targets, a Token Attention module is introduced before the third small object detection head. By tokenizing feature maps and enhancing local focusing, it enables the model to pay closer attention to small targets. Additionally, to optimize the training process, a bounding box loss function is adopted to achieve faster and more accurate bounding box predictions. YOLO-TARC simultaneously enhances the ability to retain detailed information of small targets and improves their focusing capabilities, thereby increasing detection accuracy. Experimental results on a private root canal X-ray image dataset demonstrate that YOLO-TARC outperforms other state-of-the-art object detection models, achieving a 7.5% improvement to 80.8% in mAP50 and a 6.2% increase to 80.0% in Recall. YOLO-TARC can contribute to more accurate and efficient objective postoperative evaluation of root canal treatments.

## 1. Introduction

Oral health remains a major global issue that affects quality of life. Unhealthy diets and inadequate oral hygiene have led to an increased incidence of dental diseases [1]. Bacterial infections often reach the dental pulp through caries, cracks, or periodontal diseases, causing pulpitis [2]. Root canal treatment is widely used for pulpitis. Postoperative evaluation requires periapical radiographs to assess treatment results and identify defective root canals that need repair [3]. Incomplete obturation significantly affects long-term treatment efficacy. The current analysis of the periapical radiograph relies mainly on manual observation by dentists, which is time-consuming and subjective [4]. This may lead to missed or incorrect diagnoses. Conventional dental image analysis is highly dependent on the expertise of clinicians, resulting in low efficiency, poor reproducibility, and subjective results. Delayed identification of inadequate root canal treatments postpones necessary repairs to the root canal voids.

Computer vision has made significant progress in dental image analysis [5]. Panoramic Computed Tomography (CT) has been widely used in general dental practices in developed countries. Nevertheless, in root canal therapy, periapical radiographs remain the standard to evaluate the quality of root canal obturation, while panoramic CT serves only as an auxiliary diagnostic tool when initial examinations present ambiguous findings, as described by the ESE S3-level clinical practice guidelines [6]. It should also be noted that CT is much more expensive than X-ray imaging and, in many developing countries, the accessibility of dental CT is still a challenging issue due to cost. In addition, CT and its reconstruction algorithms increase the variability in anomaly detection [7], particularly in combination with deep learning. Various methods for estimating uncertainties in medical image analysis have been explored [8,9]. However, the detection of root canal voids in X-ray images still poses challenges due to their small size, low pixel ratio, and irregular morphology. Traditional methods based on handcrafted features have limitations in adaptability and generalization. Strong background noise and complex textures often lead to false positives or missed detections. These factors make the detection of root canal voids particularly challenging in periapical X-ray images.

Among existing deep learning-based object detection algorithms, one-stage object detection algorithms, particularly the YOLOv models [10], are widely used in various detection tasks due to their fast speed, high accuracy, and ease of engineering implementation. However, YOLOv10’s approach of progressively expanding the receptive field by stacking 3 × 3 convolutional kernels results in gradual attenuation of discriminative characteristics for small targets during the feature extraction process [11].

Existing improvement methods often employ fixed-structure convolutional kernels or incorporate self-attention modules when dealing with small targets and complex scenes [12,13]. Since its introduction, the Transformer architecture has demonstrated exceptional performance in global relationship modeling through its unique self-attention mechanism [14]. However, the fully connected operations performed on each pixel during the QKV (query, key, value) projection process result in extremely high computational complexity, limiting its application in real-time object detection tasks.

To overcome these limitations, this article proposes an improved YOLOv10 network with Token Attention and Residual Convolution (YOLO-TARC) to detect small root canal voids. Specifically, we design a novel Residual Convolution (ResConv) that effectively combines the advantages of standard convolution and depthwise convolution. Through residual connections, ResConv can efficiently fuse spatially local information with cross-channel information, effectively preserving the key features of small targets. In addition, we also design a novel Tokenized Attention mechanism (TokAtt). These tokenized features not only contain local pixel information but also leverage local region tokens to focus on more detailed multiscale features. Finally, a fusion strategy is adopted to integrate global contextual information, enabling better representation of small targets and complex scenes. In summary, the main contributions of the paper are as follows:

(1) A YOLOv10-based network is proposed to integrate Token Attention and Residual Convolution (YOLO-TARC) for small void detection in root canal X-ray images, aiming to address the issues of gradual attenuation and inability to focus on the contours of small targets during feature transmission.
(2) A Residual Convolution (ResConv) is designed using residual connections to combine standard convolution and depthwise convolution. This ensures the transmission of discriminative features and effectively preserves high-frequency details at the pixel level.
(3) A novel Token Attention (TokAtt) is proposed. The input features are divided into small local region tokens. An attention mechanism is then used to dynamically adjust the weights of these tokens to focus on key information, significantly improving the ability to attend to small targets.
(4) The proposed YOLOv10-TARC is validated using a private root canal void dataset from a previous study, demonstrating superior overall performance compared to existing state-of-the-art detection methods.

## 2. Related Work

The detection of small targets has always been a challenge in object detection research [15]. In recent years, many researchers have dedicated their efforts to improving the detection performance of small targets or defects by optimizing network architectures and introducing innovative feature extraction and fusion strategies [16].

Li et al. [17] proposed UA-YOLOv5s, an adaptive small object detection algorithm based on YOLOv5s, which introduces multiscale feature fusion (MSF) technology. He et al. [18] integrated SE-CBAM into the feature extraction layer, enhancing the network’s ability to capture and utilize features. Tong et al. [19] introduced FFB to fuse deep and shallow features. Chen et al. [20] combined the Swin Transformer with convolution and introduced BRA to enhance the attention to small targets. Wen et al. [21] proposed AEFPN, which enhances FPN’s feature representation through an attention mechanism. Guo et al. [22] replaced YOLOv8s’ backbone with Faster Net, improving the feature pyramid network. Luo et al. [23] combined multiattention mechanisms to reduce missed detection of small targets. Sun et al. [24] introduced SimAM to improve feature extraction. Rehman et al. [25] proposed spatial and channel attention in a Vision Transformer for subtle architectural distortion detection in mammograms. For subtle defect detection in industrial settings, an LSTM-based deep neural network for joint spatial and temporal feature fusion was utilized for active thermography [26]. Zhorif et al. utilized the YOLOv8m object detection model with the Slicing Aided Hyper-Inference (SAHI) framework to improve detection accuracy in high-resolution aerial images [27]. However, it is not applicable to the detection of voids in dental X-ray images. In summary, current research on small object detection primarily focuses on improving network architectures, introducing new feature extraction and fusion strategies, and enhancing the model’s attention and spatial perception capabilities. These research achievements have laid a solid foundation for further improving the performance of small object detection. However, challenges remain, such as differences in resolution and semantic levels during feature fusion, the computational complexity of attention mechanisms, and the generalizability of the models.

The aforementioned work inspired us to adopt a ResConv approach to reduce differences in resolution and semantic levels during the feature fusion process, thereby effectively preserving pixel-level high-frequency details and ensuring the transmission of discriminative features such as contours of small targets during feature propagation. In addition, a TokAtt method is employed, which tokenizes feature maps and enhances internal feature focusing within tokens, thereby strengthening local focusing capabilities and improving attention to small targets, ultimately enhancing detection accuracy and robustness.

## 3. Method

**Figure 1:**
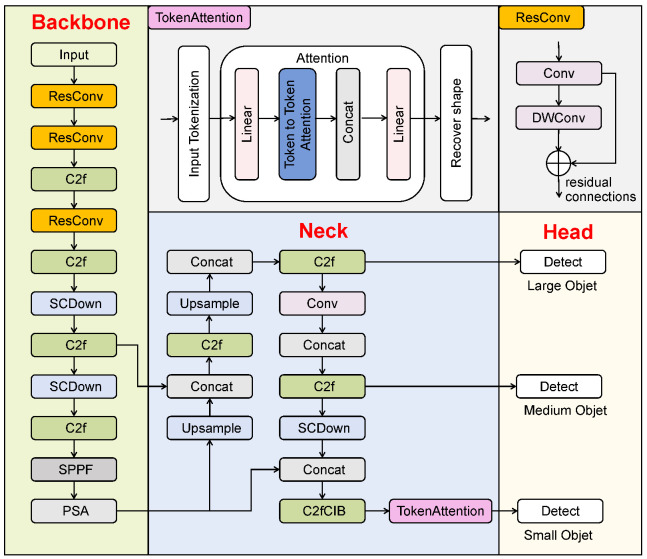
The network structure of YOLO-TARC. It mainly comprises three components: (1) the network, which includes the backbone, neck, and head; (2) ResConv for capturing contextual detail information; (3) Token Attention (TokAtt) for enhancing the model’s ability to focus on details of small targets.

We propose an improved YOLOv10 network with TokAtt and ResConv, YOLO-TARC, to detect small root canal voids in X-ray images for postoperative evaluation of root canal treatment. YOLO-TARC aims to address the issues of gradual attenuation and the inability to focus on the contours of small targets during feature transmission, enhancing attention to small targets. The YOLO-TARC network consists of four main components, input, backbone, neck, and head [10], as shown in Figure 1.

First, the input image undergoes preprocessing with mosaic high-order data augmentation and adaptive image adjustments [28,29]. The standard convolution in the backbone is replaced with the new ResConv module, which combines standard convolution (Conv) and depthwise convolution (DWConv) through residual connections, retaining the advantages of both serial and parallel connections. In the neck part, the FPN and PANet structures are inherited, effectively merging features [10]. In the head part, lightweight One-to-One and One-to-Many detection heads (large, medium, and small) are adopted, and TokAtt is placed before the third small detection head to dynamically adjust the weights of local region tokens, achieving focus on key information. Finally, the precise detection of root canal voids is achieved through the detection head.

In Section 3.1, we introduce the purpose and rationale of our new ResConv module, which expands the receptive field while preserving detailed information of small targets. In Section 3.2, we analyze the shortcomings of the neck module and introduce the TokAtt algorithm to enhance the internal feature-focusing capability of tokens by tokenizing feature maps, integrating both local and global contextual information. In Section 3.3, we improve the bounding box regression using a loss function $L_{CMIoU}$, which enables YOLO-TARC to better enhance the accuracy of tooth root canal void detection.

### 3.1. The ResConv Module

**Figure 2:**
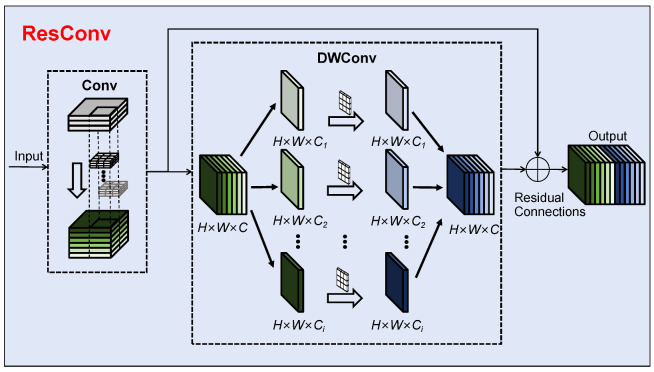
Structurediagram of ResConv module, which combines standard convolution (Conv) and depthwise convolution (DWConv) through residual connections.

To better leverage the receptive field expansion effect of deep features while preserving the detailed information of small targets for detecting root canal voids, we redesign the convolutional block. The ResConv module aims to address the limitations of the original standard convolution in YOLOv10, such as global information capture and fixed sampling shapes, which affect the extraction and representation of deep features for small targets. Unlike other convolutional design approaches, ResConv is inspired by the residual connections in ResNet [30] by redesigning the convolutional module. It introduces residual connections into the design of convolutions. The structure of the ResConv module is shown in Figure 2. The core idea of ResNet is residual connectivity. With residual connections, the network can bypass certain layers via skip connections, directly passing information from one layer to subsequent layers [30]. To combine the advantages of Conv and DWConv [31], unlike other approaches that use purely serial or parallel connections, this paper redesigns the ResConv module, drawing inspiration from residual connections. As shown in Figure 2, the ResConv module connects Conv and DWConv through residual connections while retaining the benefits of both serial and parallel connections. It allows discriminative features such as the edge contours of small targets to be transmitted during feature propagation, further enhancing the network performance in small target detection.

### 3.2. The TokAtt Module

The YOLOv10 network introduces a C2fCIB before the third detection head in the neck part. Although C2fCIB can enhance feature extraction capabilities, it lacks sufficient focus on fine and important features in the detection of small targets against a complex background, affecting detection accuracy.

**Figure 3:**
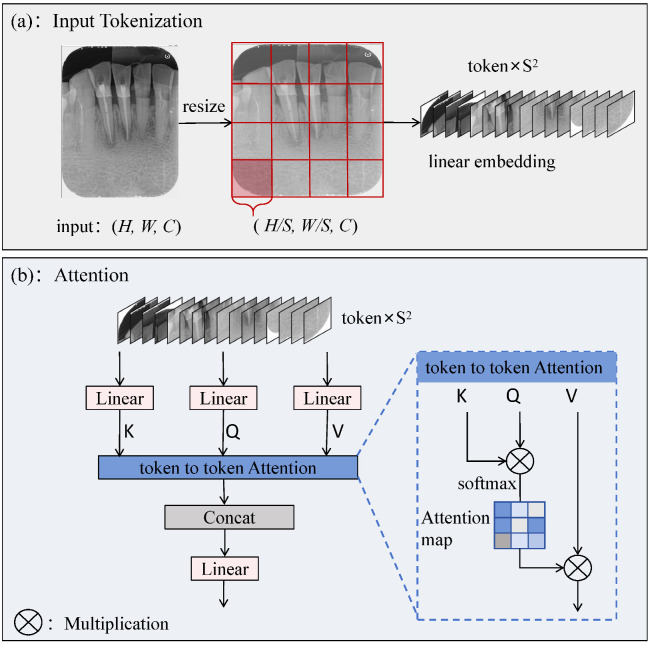
The Token Attention (TokAtt) mechanism. (**a**) Input tokenization. The input tensor (H,W,C) is divided into $S^{2}$ tokens, with each token having dimensions of (H/S,W/S,C). (**b**) Attention. It inputs the $S^{2}$ tokens into the token-to-token Attention module, utilizing attention scores K, Q, V to adjust the importance of each token.

To address this issue, some researchers have proposed sparse attention mechanisms [32,33], where each query only focuses on a small number of pairs of key values rather than all of them. However, these approaches either use manually created static patterns or share a subset of key–value pairs across all queries. We introduce the TokAtt module at the end of the C2fCIB module, as shown Figure 3.

Inspired by the Vision Transformer (ViT), the TokAtt mechanism is redesigned, drawing on the idea of multihead attention [14]. The core idea is to divide the input image into local tokens and then perform token attention calculations for each local token separately. This allows for more accurate attention to the information within each input token, achieving focus on key information, and finally fusing all tokens.

First, the feature map is tokenized. Given a 2D input feature map $X\in R^{H\times W\times C}$, we first divide it into nonoverlapping token regions $S^{2}$, as shown in Figure 3a. The input image is resized to 640×640 to ensure that it can be evenly divided into $S^{2}$ tokens of equal size, followed by a linear embedding operation on the tokens. Next, the internal feature-focusing capability of the tokens is enhanced. The specific operation is shown in Figure 3b. Linear operations are performed on queries, keys, and values, followed by token-to-token attention for each local token. Finally, each token is reshaped to its original shape (H,W,C) through a fusion strategy, followed by a linear operation.

Specifically, with queries $Q\in R^{N_{q}\times C}$, keys $K\in R^{N_{kv}\times C}$, and values $V\in R^{N_{kv}\times C}$ as inputs, the attention function transforms each query into a weighted sum of values, where the weights are computed as the normalized dot product between the queries and the corresponding keys. It can be defined in matrix form as

(1) $$\begin{array}{c} \mathrm{Attention}\left(Q,K,V\right)=\mathrm{Softmax}\left(\frac{QK^{T}}{\sqrt{C}}\right)V, \end{array}$$

where the scaling factor $\sqrt{C}$ is introduced to avoid concentrated weights and gradient vanishing [32].

To simplify notation, we discuss the case of single-head self-attention with a single input. In Algorithm 1, TokAtt is summarized using pseudocode.
**Algorithm 1** Pseudocode of TokAtt mechanism.0:Input: Features (H,W,C). Resize input, assuming H==W.0:Output: Features (H,W,C).1:**1.Tokenize input (H,W,C) into (H/S,W/S,C).**2:S←numberofregions,3:x←tokenize(input,tokensize=S).4:**2.Attention**.5:Q,K,V←Linear(x),6:$\mathrm{Attention}\;\mathrm{Weights}\leftarrow \mathrm{Softmax}\left(\frac{QK^{T}}{\sqrt{c}}\right)$,where $\sqrt{c}$ is a scaling factor,7:Output←AttentionWeights×V.8:**3.Recover shape to (H,W,C).**9:Output←untokenize(output,tokensize=H/S).

After integrating TokAtt, the C2fCS module dynamically adjusts attention during feature fusion, enhancing the network’s focus on key image regions and target details.

### 3.3. The Bounding Box Loss Function

The loss function of YOLOv10 consists of three components:

(2) $$\begin{array}{c} L=L_{\mathrm{dff}}+L_{\mathrm{cls}}+L_{\mathrm{box}}, \end{array}$$

where $L_{dff}$ is the loss of localization for offsets, $L_{cls}$ the loss of classification for categories, and $L_{box}$ the regression loss for the predictions of the bounding box, which is used to measure the positional difference between the predicted bounding boxes and the ground truth boxes [10].

YOLOv10 employs the CIoU loss to compute $L_{box}$ It is defined as follows:

(3) $$\begin{array}{c} L_{CIoU}=1-IoU+\frac{\rho^{2}\left(b,b^{gt}\right)}{c^{2}}+\alpha v, \end{array}$$

(4) $$\begin{array}{c} \alpha =\frac{v}{(1-IoU)+v}, \end{array}$$

(5) $$\begin{array}{c} v=\frac{4}{\pi^{2}}{\left[arctan\left(\frac{w^{gt}}{h^{gt}}\right)-arctan\left(\frac{w}{h}\right)\right]}^{2}, \end{array}$$

where IoU represents the Intersection over Union between the predicted and ground truth boxes; $\rho^{2}\left(b,b^{gt}\right)$ denotes the squared Euclidean distance between the centers of the predicted and ground truth boxes; *b* and $b^{gt}$ are the coordinates of the center points of the predicted and ground truth boxes; *c* is the diagonal distance of the minimum bounding box; α is a weighting factor used to balance the weights between the different loss terms; *v* is a penalty term used to further adjust the loss function; $w^{gt}$ and $h^{gt}$ are the width and height of the ground truth box; and *w* and *h* the width and height of the predicted box.

**Figure 4:**
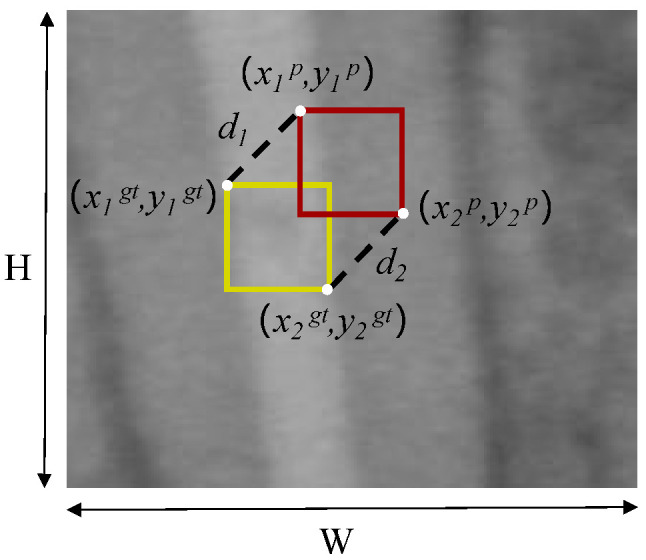
Illustration of the MPDIoU loss function. *H* and *W* are the height and width of the image. The yellow rectangle denotes the ground truth box and the red one the predicted box. $\left(x_{1}^{gt},y_{1}^{gt}\right)$ and $\left(x_{2}^{gt},y_{2}^{gt}\right)$ are the coordinates of the top-left and bottom-right corners of the ground truth box, respectively, while $\left(x_{1}^{p},y_{1}^{p}\right)$ and $\left(x_{2}^{p},y_{2}^{p}\right)$ are the coordinates of the top-left and bottom-right corners of the predicted box, respectively.

We employ a new bounding box regression loss function, MPDIoU [34], to replace IoU. The MPDIoU loss function is illustrated in Figure 4.

The MPDIoU loss function is defined as follows:

(6) $$\begin{array}{c} L_{MPDIoU}=IoU-\frac{d_{1}^{2}+d_{2}^{2}}{H^{2}+W^{2}}, \end{array}$$

(7) $$\begin{array}{c} d_{1}^{2}={\left(x_{1}^{p}-x_{1}^{gt}\right)}^{2}+{\left(y_{1}^{p}-y_{1}^{gt}\right)}^{2},\;d_{2}^{2}={\left(x_{2}^{p}-x_{2}^{gt}\right)}^{2}+{\left(y_{2}^{p}-y_{2}^{gt}\right)}^{2}, \end{array}$$

where $d_{1}^{2}$ and $d_{2}^{2}$ represent the squared Euclidean distances between the top-left and bottom-right corners of the ground truth box and the predicted box, respectively.

A new loss function $L_{CMIoU}$ is defined as

(8) $$L_{CMIoU}=1-IoU+\frac{d_{1}^{2}+d_{2}^{2}}{H^{2}+W^{2}}+\frac{\rho^{2}\left(b,b^{gt}\right)}{c^{2}}+\alpha vs.,$$

It effectively addresses the issue of zero gradients when the predicted and ground truth boxes do not overlap, ensuring that the model can still optimize parameters through gradient descent.

## 4. Experiments

We evaluated the effectiveness of our proposed YOLO-TARC. First, we introduce the experimental details, including dataset preparation, experimental environment, and evaluation metrics (Section 4.1). The comparative experiments demonstrate that the proposed method outperforms existing techniques in terms of detection accuracy (Section 4.2). Additionally, to verify the effectiveness of the proposed ResConv, TokAtt, and other structural design choices, we conducted ablation studies (Section 4.3). The ablation studies show that the ResConv and TokAtt modules enhance the detection performance of small targets and significantly improve accuracy in detecting small root canal voids. Finally, to verify that ResConv effectively preserves pixel-level high-frequency details through its residual connections, and that the discriminative features of small targets are transmitted during the feature propagation process, we visualize the attention maps.

### 4.1. Implementation Details

**Dataset:** The root canal void dataset used in this study consists of 768 dental X-ray images collected by a hospital clinic after root canal treatment, as described in a previous study, where institutional ethics approval was obtained before the study and each volunteer was informed of and agreed to the purpose and procedure of the study when they came to the clinic for dental examinations and root canal treatments, and they signed individual consent forms [28]. The dataset is randomly divided into two subsets: 80% (612 samples) for training and 20% (156 samples) for validation.

**Experiment settings:** The experiments were conducted using the AdamW optimizer for training, with the learning rate set to $10^{-2}$. The training batch size is 16, the initial learning rate is 0.01, the momentum parameter is 0.937, and the weight decay coefficient is 0.0005. To ensure fairness, all experiments were conducted under consistent conditions (hardware: NVIDIA RTX 4090 GPU; software: PyTorch 2.3.0, CUDA 12.1, Python 3.10).

**Evaluation metrics:** To be consistent with other studies, we use Recall, Precision, and mean Average Precision (mAP50% and mAP50:95%) as the primary detection metrics for quantitative evaluation. Their calculations are shown in Equations (9) and (10).

(9) $$\begin{array}{c} Recall=\frac{TP}{TP+FN},\;Precision=\frac{TP}{TP+FP}, \end{array}$$

where TP represents the number of true positives correctly predicted by the model, FN the number of false negatives, and FP the number of false positives.

(10) $$\begin{array}{c} AP=\int_{0}^{1}P\left(R\right)\;dR,\;mAP=\frac{\sum_{i=1}^{N}AP_{i}}{N}, \end{array}$$

where AP is calculated as the area under the Precision–Recall (PR) curve for a single category. mAP is the average of AP values across all categories, where N is the total number of categories in the detection task. In our experiments, we specifically focus on measuring mAP50 and mAP50-95.

### 4.2. Comparison Experiments

To validate the performance of YOLO-TARC in detecting small root canal voids, under the same experimental conditions and dataset, we selected the latest object detection algorithm, YOLOv10m, as well as mainstream object detection models such as Faster R-CNN [35], SSD [36], YOLOv5 [37], YOLOv7-tiny [38], YOLOv8m [39], YOLOv9m [40], YOLOv10m [10], and YOLOv11m [41].

As shown in Table 1, compared to other models, the proposed YOLO-TARC demonstrates significant advantages in recall, precision, mAP50, and mAP50-95. It achieves the highest recall among the nine models, outperforming the popular YOLOv8m, YOLOv9m, YOLOv10m, and YOLOv11 by 12.4%, 10.4%, 6.2%, and 15.7%, respectively. It also shows substantial improvements in mAP50 compared to the other eight models, outperforming YOLOv8m, YOLOv9m, YOLOv10m, and YOLOv11 by 10.9%, 9.0%, 7.5%, and 8.8%, respectively. As an important metric for evaluating object detection algorithms, mAP50 reflects the accuracy and robustness of the model in detecting small root canal voids.

**Figure 5:**
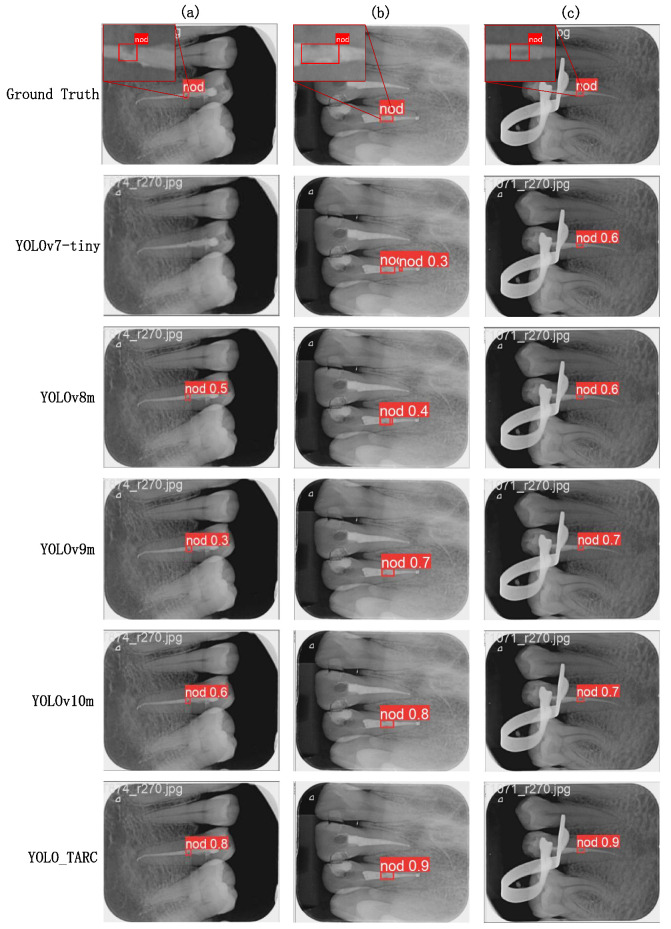
Visualization of detection results for four instances. (**a**) The void is located on the side of the root canal. (**b**) The void is relatively large and the background is blurred. (**c**) The void is located in the middle of the root canal, and the image is clear.

To visually demonstrate the detection performance of YOLO-TARC, we compared it to five other models with relatively high average precision, as shown in Figure 5.

In Figure 5a, the void is on the side of the root canal, and other methods may miss the detection or have low detection accuracy. YOLO-TARC detects the root canal void on the side very well. In Figure 5b, the root canal void is large and the background is blurred, leading to poor performance by YOLOv7-tiny and YOLOv8m. In Figure 5c, the image is clear and the contrast is obvious, which allows all methods to detect the root canal void correctly.

### 4.3. Ablation Experiments

**Effects of network components:** To evaluate the effectiveness of the improvement modules proposed in this study, ablation experiments were conducted on the YOLOv10m baseline model to evaluate the contributions of each module in YOLO-TARC. The results are shown in Table 2.

The first row shows YOLOv10m as the baseline model. The second row presents the results after replacing with ResConv, where the most notable improvement is precision, reaching 82.4%, a 5% increase over the baseline model. The results indicate that ResConv effectively addresses the issue of small target loss by preserving detailed information in deep feature maps, thereby improving Precision. The third row shows the results after introducing TokAtt, with Recall, Precision, mAP50, and mAP50-95 increasing by 3.7%, 4.1%, 4.7%, and 2.8%, respectively. The TokAtt enhances the ability to focus on local information. The fifth row presents the results of replacing ResConv with TokAtt. Compared to the baseline model, Recall, Precision, and mAP50 increased by 5.8%, 3%, and 5.9%, respectively. Although the 3% improvement in Precision is not as significant as the previous 5%, the Recall and mAP50 showed notable improvements after the introduction of TokAtt. For root canal void detection, reducing missed detections is more critical, so the focus is more on improving Recall while balancing Precision and Recall. The results indicate that introducing the TokAtt that focuses on local small targets can further enhance the model’s performance in small root canal void detection.

The last row in Table 2 is based on the introduction of $L_{CMIoU}$ on the foundation established above as the full model, achieving the best performance in Recall and mAP50 among all ablation configurations. Compared to the baseline model, Recall, Precision, mAP50, and mAP50-95 increased by 6.2%, 2.1%, 7.5%, and 2.8%, respectively. This shows that the model’s ability to detect small and void regions, especially in the evaluation of root canal filling quality, has been significantly enhanced when dealing with complex target areas.

**Figure 6:**
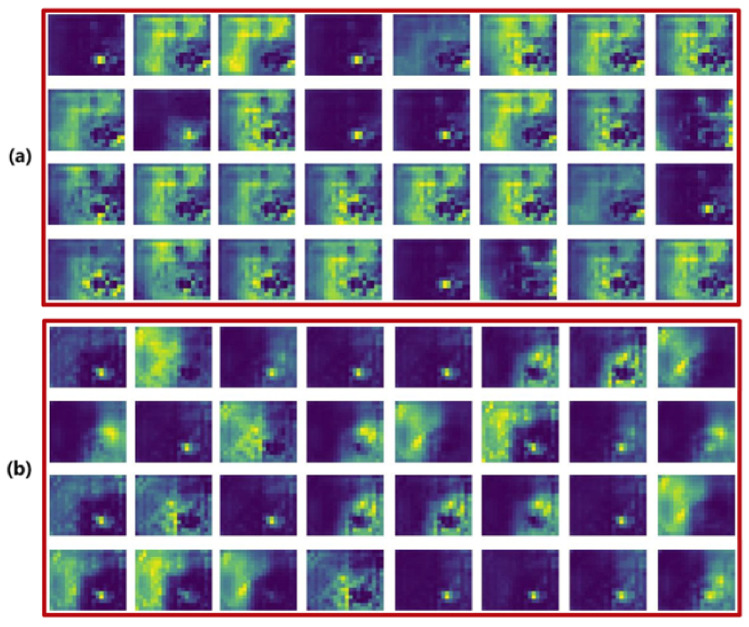
Visualization of backbone feature maps. (**a**) YOLOv10m. (**b**) YOLO-TARC.

**Effects of ResConv:** To verify whether residual connections between Conv and DWConv can effectively preserve high-frequency details at the pixel level and ensure transmission of discriminative characteristics, such as small target contours, during feature propagation, we visualized the feature maps output by the YOLOv10m baseline and YOLO-TARC backbones, respectively. As shown in Figure 6, the feature maps of YOLO-TARC preserve more regions of small targets. This confirms that the ResConv module effectively preserves the features of small target regions.

## 5. Conclusions and Future Work

Detecting root canal voids is a crucial task for objective postoperative evaluations of root canal treatment. We propose an improved YOLOv10, termed YOLO-TARC, which incorporates Token Attention (TokAtt) and Residual Convolution (ResConv), specifically for the detection of small root canal voids. To address the existing models’ inability to effectively retain key features of small objects and their weak focusing capabilities, several improvements were implemented. First, ResConv is used to ensure the transmission of discriminative features such as the contours of small objects during the feature propagation process. Second, TokAtt is introduced before the third small object detection head to enhance the attention given to small objects. Furthermore, finally, a new loss function is utilized. Experimental results show that YOLO-TARC achieves a 7.5% improvement to 80.8% in mAP50 and a 6.2% increase to 80.0% in Recall compared to state-of-the-art models, not only improving detection accuracy but also reducing missed detections. These improvements in YOLO-TARC not only provide an effective solution to detect small root canal voids but also offer new possibilities for other small object detection applications. We plan to test the robustness of the model in more complex scenarios, such as multiobject detection tasks and multimodal tasks, in the future.

## Figures and Tables

**Table 1:** Performance results of different detection algorithms on the root canal void dataset.

Method	Recall (%)	Precision (%)	mAP50 (%)	mAP50-95 (%)
Faster R-CNN [35]	59.8	62.8	61.7	24.1
SSD [36]	58.8	61.6	56.1	18.2
YOLOv5 [37]	61.3	70.7	65.9	30.7
YOLOv7-tiny [38]	62.5	63.6	64.5	25.6
YOLOv8m [39]	67.6	74.2	69.9	31.6
YOLOv9m [40]	69.6	78.1	71.8	34.0
YOLOv10m (baseline) [10]	73.8	77.4	73.3	34.3
YOLOv11 [41]	64.3	78.0	72.0	31.7
YOLO-TARC (ours)	**80.0**	**79.5**	**80.8**	**37.1**

Bold numbers represent the highest values in each column.

**Table 2:** Results of ablation experiments.

YOLOv10m	ResConv	TokAtt	Loss $L_{\mathrm{CMIoU}}$	Rec (%)	Pre (%)	mAP50 (%)	mAP50-95 (%)
✔				73.8	77.4	73.3	34.3
✔	✔			73.5	**82.4**	75.3	36.5
✔		✔		77.5	81.5	78.0	37.1
✔			✔	72.9	78.3	74.5	34.5
✔	✔	✔		79.6	80.4	79.4	**37.3**
✔		✔	✔	77.9	81.7	78.7	36.9
✔	✔		✔	74.7	81.5	76.0	36.2
✔	✔	✔	✔	**80.0**	79.5	**80.8**	37.1

Rec: Recall, Pre: Precision. ✔ indicates that the corresponding module is included in the experiment. Bold numbers represent the highest values in each column.

## Data Availability

The data that support the findings of this study can be obtained from the corresponding authors upon reasonable request.
